# Mediation of BMI on 25-Hydroxyvitamin D Levels in U.S. Adults with Sugar-Sweetened Beverages Consumption

**DOI:** 10.3390/nu15153291

**Published:** 2023-07-25

**Authors:** Wei-Ting Lin, Gabrielle V. Gonzalez, Yu-Hsiang Kao, Hui-Yi Lin, Mirandy S. Li, David W. Seal, Chien-Hung Lee, Chih-yang Hu, Lei-Shih Chen, Tung-Sung Tseng

**Affiliations:** 1 Social, Behavioral, and Population Sciences, Tulane University School of Public Health and Tropical Medicine, New Orleans, LA 70112, USA; wtlin0123@gmail.com (W.-T.L.); dseal@tulane.edu (D.W.S.); 2 Behavioral and Community Health Sciences Program, School of Public Health, Louisiana State University Health Sciences Center, New Orleans, LA 70112, USA; ggonz4@lsuhsc.edu (G.V.G.); moroskao@gmail.com (Y.-H.K.); mli2@lsuhsc.edu (M.S.L.); 3 Biostatistics Program, School of Public Health, Louisiana State University Health Sciences Center, New Orleans, LA 70112, USA; hlin1@lsuhsc.edu; 4 Department of Public Health, College of Health Science, Kaohsiung Medical University, Kaohsiung 80708, Taiwan; cnhung@kmu.edu.tw; 5 Research Center for Environmental Medicine, Kaohsiung Medical University, Kaohsiung 80708, Taiwan; 6 Environmental and Occupational Health Sciences, Louisiana State University Health Sciences Center, New Orleans, LA 70112, USA; chu@lsuhsc.edu; 7 Department of Health and Kinesiology, Texas A&M University, College Station, TX 77843, USA; lace@hlkn.tamu.edu

**Keywords:** sugar-sweetened beverage (SSB), body mass index (BMI), 25-hydroxyvitamin D (25(OH)D), vitamin D deficiency, NHANES

## Abstract

Body mass index (BMI) as well as sugar-sweetened beverages (SSB) has been suggested to independently decrease 25-hydroxyvitamin D (25(OH)D). However, the relationship between SSB, BMI, and 25(OH)D is uncertain. This study aimed to investigate the potential mediating role of BMI in the association between SSB intake and 25(OH)D. A total of 4505 representative U.S. adults aged above 20 years and without liver conditions were selected from the 2013–2014 NHANES. All analyses were performed under survey modules with appropriate sampling weights. The prevalence of 25(OH)D insufficiency and deficiency was 37.8% and 24.1% in U.S. adults, respectively. Compared with non-SSB consumers, an increased risk of vitamin D deficiency was found in either heavy SSB consumers or soda consumers, respectively (aOR = 2.10, 95% CI = 1.25–3.54 in heavy SSB consumers; aOR = 1.61, 95% CI = 1.06–2.44 in soda consumers). Around 21.3% of the total effect of sugar intake from SSB on decreased 25(OH)D was explained by BMI. In conclusion, high total sugar intake from SSB and BMI independently contribute to lower 25(OH)D, and BMI mediates the inverse association between total sugar intake from SSB intake and 25(OH)D. Furthermore, an increased risk of having vitamin D deficiency was found in the population who consumed higher levels of sugar from SSB or soda drinks.

## 1. Introduction

Vitamin D, a fat-soluble vitamin found in foods, is commonly added to some foods and used as a dietary supplement [1]. Vitamin D is an essential nutrient and maintains bone mineral density and homeostasis of calcium and phosphorus [2]. 25-hydroxyvitamin D (25(OH)D) is a commonly used biomarker for vitamin D status [3]. Accumulating studies have shown that low concentrations of 25(OH)D are associated with numerous health conditions, including bone health [4], glucose homeostasis [5], development of hypertension and cardiovascular disease [6], cancer-related mortality and progression [7], depression [8], inflammation [9], multiple sclerosis [10], and metabolic syndrome [11]. Based on clinical recommendations, individuals with serum 25(OH)D levels ≤ 20 ng/mL and 21–29 ng/mL are defined as having vitamin D deficiency and insufficiency, respectively [3,12]. Results from the 2001–2010 U.S. nationwide survey showed that 28.9% and 41.4% of U.S. adults were vitamin-D-deficient and -inadequate, respectively [13]. A literature review also indicated that the global prevalence of vitamin D deficiency is around one billion [14]. In addition to exposure to sunlight, diet, and supplement intake, several factors may also be related to the vitamin D insufficiency or deficiency, including age, ethnicity, lifestyle behaviors, socioeconomic status, and adiposity status [13,15,16].

Obesity is a well-known contributor to several health outcomes [17]. Findings from a U.S. population-based survey during 2017 and 2018 reported that 42.4% of adults aged 20 and above were obese [18]. Recently, accumulating studies have focused on exploring the association between obesity and serum 25(OH)D status. A 3.4 nmol/L decreased serum 25(OH)D concentration was found in individuals who were obese compared to those who were normal weight, even after adjusting for sociodemographic factors, lifestyle patterns, and metabolic-related components [16]. A meta-analysis study suggested that obesity may be a risk factor for lower 25(OH)D levels. Findings showed that 1.15% decreased 25(OH)D was estimated in each unit of increased BMI (1 kg/m^2^). In contrast, a causal effect of low 25(OH)D levels on increased BMI was not detected [19]. Not only is obesity a well-known global epidemiological issue, but vitamin D deficiency has also been noticed and recognized as a public health problem worldwide [19,20].

According to the 2010 and 2015 National Health Interview Survey (NHIS), 63.0% of U.S. adults self-reported consumption of sugar-sweetened beverages (SSB) at least one time every day [21]. SSB consumption, which has a high amount of added sugar and zero nutrients, has been strongly related to obesity, hyperuricemia, inflammation, and metabolic-related outcomes [22,23,24]. There are reports of negative associations between SSB consumption and 25(OH)D concentrations, but the reason why is still elusive [25,26,27]. Studies reporting an association cite issues with understanding the biological mechanism behind the association, such as the possible role of fructose [25] and obesity level in children [26]. Additionally, one study found a minimal positive association between 25(OH)D concentrations and non-alcoholic drinks [27].

The previously discussed studies found significant negative associations between SSB consumption and 25(OH)D concentrations, but these studies were limited in sample size and population type. Furthermore, data on SSB consumption in these previous studies were only obtained from a food frequency questionnaire (FFQ) that could not provide quantity-related indicators from SSB. More studies are needed in the general population to understand why we are seeing cases of significant negative associations between amounts of total sugar intake from SSB and certain types of SSBs and 25(OH)D and if these findings also translate to larger, more representative populations. Moreover, the relationship between SSB consumption, BMI, and 25(OH)D levels is limited. The aim of the present study was (1) to evaluate the association between SSB consumption, BMI, and risk of vitamin D insufficiency and deficiency and (2) to determine whether BMI is a mediator that contributes to the association between SSB consumption and lower 25(OH)D levels.

## 2. Materials and Methods

### 2.1. Study Population

The current study is an observational, retrospective study design based on a secondary analysis of the National Health and Nutrition Examination Survey (NHANES) data from the 2013–2014 cycle. The NHANES, a nationally representative sample of the U.S. population based on a complex, multistage, and stratified design, is a program of studies conducted every two years by the Centers for Disease Control (CDC) and Prevention of the National Center for Health Statistics (NCHS) to assess the health and nutritional status of adults and children in the U.S. Information on demographics, health-related questionnaires, dietary interviews, BMI, biochemical examination, and 25-hydroxyvitamin D in the cycle from 2013–2014, which are the publicly available data, were utilized to explore the current study purposes. The detailed study design, methods, and data collection of the NHANES are available elsewhere [28] Information from each publicly available set of data was combined and appropriately transformed to the final analyzed dataset. A total of 4505 adults without diagnosed liver conditions and who were aged ≥20 years old were selected into analysis. This project was reviewed and approved by the NCHS Ethics Review Board. Written informed consent was obtained from each NHANES participant.

### 2.2. Questionnaires

Demographic data including age, gender, ethnicity, and socio-economic status were collected by trained interviewers using the computer-assisted personal interview (CAPI) system. The ratio of family income to poverty (PIR) was calculated based on total family income and poverty threshold. Lifestyle patterns such as cigarette smoking, alcohol drinking, physical activity, and self-reported personal medical conditions were obtained from each section of the Mobile Examination Center (MEC) questionnaire. The MEC questionnaire was conducted by a trained MEC interviewer. Two questions from the cigarette-use section, i.e., “Have you smoked at least 100 cigarettes in your life?” and “Do you now smoke cigarettes?”, were used to identify cigarette-use status. First, NHANES participants who answered “no” to the question “Have you smoked at least 100 cigarettes in your life?” were classified into the non-smoker category. Then, we used the second question, namely “Do you now smoke cigarettes?” to identify a former smoker if the NHANES participant answered “not at all” and a current smoker if the NHANES participant answered “some days” or “every day”. NHANES participants who consumed <12 alcoholic drinks in the past year were defined as non-alcohol-drinkers. Individuals who consumed ≥12 alcoholic drinks in the past year and ≤5/>5 alcoholic drinks on a given day were defined as light/heavy alcohol drinkers. The status of weekly physical activity was calculated based on the type of recreational activities (moderate and vigorous intensity) and duration of activities in leisure time. Adequate or insufficient physical activity per week was defined according to World Health Organization (WHO) recommendations [29]. Self-reported health/medical history, including asthma, arthritis, hypertension, heart diseases/attack, stroke, diabetes, angina, emphysema, thyroid problem, chronic bronchitis, and cancer/malignancy, was obtained in the medical conditions’ questionnaire section and considered as potential confounders in this study.

### 2.3. Dietary Intake

Data on daily consumption of energy (kcal), sugar (g), vitamin D (D2 + D3) (μg), and calcium (mg) in the diet were obtained from two 24-h dietary recall interviews. The first interview was conducted in-person at the MEC. Then, 3 to 10 days after the first interview, the second interview was conducted by telephone. An average total daily energy and total daily sugar consumption in the diet were calculated if NHANES participants completed two 24-h dietary recall interviews, respectively. Intake of vitamin D (D2 + D3) (μg) and calcium (mg) from supplements in the past 30 days was combined with daily vitamin D (D2 + D3) (μg) and calcium (mg) intake in diet. According to the recommended dietary allowances (RDAs) for adults, the adequate intake (AI) for vitamin D is 15 μg and 20 μg for adults aged ≤70 and ≥71 years, respectively. The AI for calcium is 1000 mg for women aged 19–50 years and 1200 mg for women ≥51 years old. For men aged 19–70 and ≥71 years, the AI for calcium is 1000 mg and 1200 mg, respectively [30].

### 2.4. SSB Intake

We included regular soda drinks, non-100% juice, juice-flavored sweetened drinks, and tea/coffee with added sugar in this study. Detailed data on daily consumption of sugar from each type of SSB were obtained from the dietary interview component based on the United States Department of Agriculture (USDA) Food and Nutrient Database for Dietary Studies (FNDDS). Total added sugar intake on a given day from each type of SSB was aggregated. On average, approximately 40–50 g sugar was estimated in one can/serving of soda in the U.S, [31]. We further categorized total added sugar intake from SSB into <40, 40–79, and ≥80 g categories. In order to consider the effect of high amounts of high-fructose corn syrup (HFCS) in regular soda, we further classified SSB consumers into non-soda SSB consumers and soda consumers [32]. Individuals who only consumed non-100% juice, juice-flavored sweetened drinks, and tea/coffee were defined as non-soda SSB consumers. Individuals who consumed soda drinks only or both soda and non-soda SSB were defined as soda consumers. Non-SSB consumers were defined as subjects not consuming any type of SSB [33].

### 2.5. Body Mass Index

Status of body adiposity was identified by using BMI from the body measures data of the examination section. We used the Centers for Disease Control and Prevention (CDC) suggested cut-off value, which is set as <25, 25–29.9, and ≥30 kg/m^2^ for defining adult normal weight, overweight, and obesity, respectively [34].

### 2.6. Clinical Examination

Serum 25-hydroxyvitamin D (25(OH)D) measurements in NHANES laboratory files were used in this study. 25(OH)D concentrations were performed at the National Center for Environmental Health, CDC, Atlanta, GA using the DiaSorin RIA kit (Stillwater MN), using a standardized liquid chromatography–tandem mass (LC-MS/MS) method [35]. We further categorized continuous 25(OH)D values into normal, vitamin-D-insufficient, and vitamin-D-deficient if individuals had 25(OH)D values >30, 20–29, and <20 ng/mL, respectively [36].

Data on total triglycerides, alkaline phosphatase, and phosphorus on 25(OH)D were obtained from the Standard Biochemistry Profile in NHANES laboratory files. Following the American Heart Association (AHA) criteria, subjects who had total triglycerides levels of 150 mg/dL or higher were defined as having elevated total triglycerides [37]. In adults, normal concentrations of alkaline phosphatase (ALP) and phosphate ranged from 20 to 140 and 2.5 to 4.5 mg/dL in serum, respectively [38,39,40].

### 2.7. Statistical Analysis

All statistical analyses were performed under survey modules with an appropriate sampling weight using STATA v17 (StataCorp., College Station, TX, USA). The descriptive results are presented as percentages under chi-square test for categorical variables and mean with standard errors (mean ± se) under simple linear regression models for continuous variables. Multivariate-adjusted differences were used to assess the association between sugar intake from total SSB or type of SSB intake and BMI values and 25(OH)D concentrations under multivariable regression models, respectively. A mediation analysis was performed to understand whether BMI plays a mediator in the association between total sugar intake from SSB and 25(OH)D concentrations. The total effect, the direct effect, and the indirect effect were measured in the mediation analysis, and the proportion-mediated effect of BMI was further estimated. A multinominal-logistic-regression-models-derived odds ratio (OR) with 95% confidence intervals (CI) was conducted to evaluate the effect of sugar intake from all SSB or type of SSB consumption the vitamin D insufficient and deficiency.

## 3. Results

Table 1 presents the sampling-weights-adjusted distribution of demographic factors and lifestyle patterns among 25(OH)D status. The prevalence of vitamin D insufficiency and deficiency was 37.8% and 24.1% in U.S. adults, respectively. A higher prevalence of vitamin D deficiency was found in individuals who were male, non-Hispanic Black, below poverty, current smokers, physical activity inactivity, and without self-reported medical conditions (all *p* ≤ 0.002).

The sampling-weights-adjusted distribution of dietary patterns and SSB-related factors, BMI, and biomedical examination among 25(OH)D status is demonstrated in Table 2. The daily total energy and total sugar intake were not associated with 25(OH)D levels. In total, 31.6% and 29.3% of individuals with vitamin D deficiency consumed insufficient vitamin D and calcium intake from dietary and supplements, respectively (all *p* < 0.001). Individuals had high added sugar intake from SSB also tended to be vitamin-D-deficient (*p* < 0.001). Among SSB consumers, the prevalence of vitamin D deficiency was significantly higher in soda consumers than in non-soda consumers (32.1% in soda consumers and 23.3% in non-soda consumers, *p* = 0.002). Obese individuals were more likely to be vitamin-D-deficient (*p* < 0.001).

Potential confounders were firstly selected from Table 1 and Table 2, which were significantly associated with 25(OH)D status, such as age, gender, ethnicity, family income, cigarettes, physical activity status, personal medical conditions, and daily total intake of vitamin D and calcium. Second, alcohol use and daily total intake of energy and sugar were also considered as potential confounders based on previous findings’ suggestions.

The multivariate-adjusted association between total sugar intake from SSB, BMI, and 25(OH)D levels is shown in Table 3. When compared to non-SSB consumers, individuals who consumed 40–79 and ≥80 g of sugar intake from total SSB per day had a 1.83–2.88 ng/mL lower level of 25(OH)D after adjusting for demographic characteristics, lifestyle factors, and dietary pattern. However, a significantly lower 25(OH)D level was no longer found in individuals who consumed 40–79 g of sugar from SSB compared to non-SSB consumers after additionally adjusting for BMI. A 2.53 ng/mL lower level of 25(OH)D was still observed in U.S. adults who consumed ≥80 g of sugar from total SSB when compared to non-SSB consumers, even after additionally adjusting for BMI status (95% CI = −3.93, −1.12). Table 4 presents the multivariate-adjusted association between different type of SSB, BMI, and 25(OH)D levels. A significantly decreased level of 25(OH)D was only observed in soda consumers when compared to non-SSB consumers after adjusting for all potential confounders (adjusted difference = −1.92; 95% CI = −3.14, −0.70).

**Figure 1:**
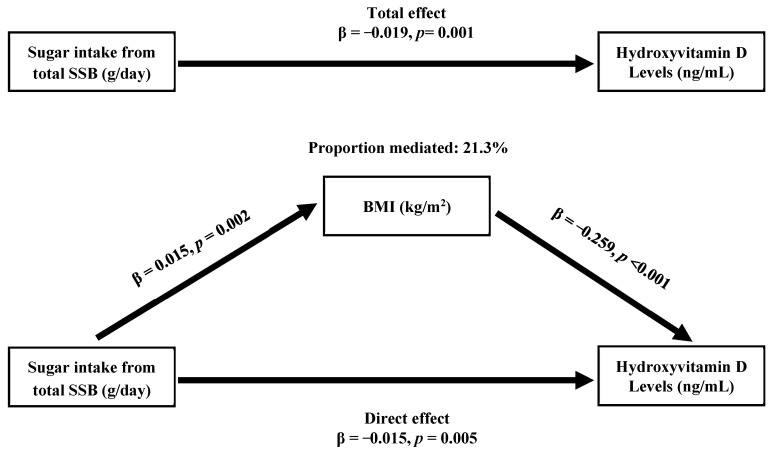
Body mass index (BMI) mediates the association between sugar intake from total sugar-sweetened beverage (SSB) and serum hydroxyvitamin D levels. Potential confounders included demographic factors, cigarettes and alcohol use, physical activity status, personal medical conditions, and daily total intake of energy, sugar, vitamin D, and calcium; these were adjusted for in mediation analysis.

A mediation analysis was performed to understand the role of BMI in the association between consumption of sugar from total SSB and 25(OH)D levels. In the mediation model, demographic factors, lifestyle, and dietary pattern were considered as potential confounders, including age, gender, ethnicity, PIR, status of cigarettes and alcohol use, physical activity, medical conditions, and total daily consumption of energy, sugar, vitamin D, and calcium. The total effect on the 25(OH)D was −0.019 (*p* = 0.001). The direct effect and indirect effects were −0.015 and −0.004, respectively (*p* ≤ 0.011). Approximately 21.3% of the effect of total sugar intake from SSB on was mediated by BMI (Figure 1).

**Figure 2:**
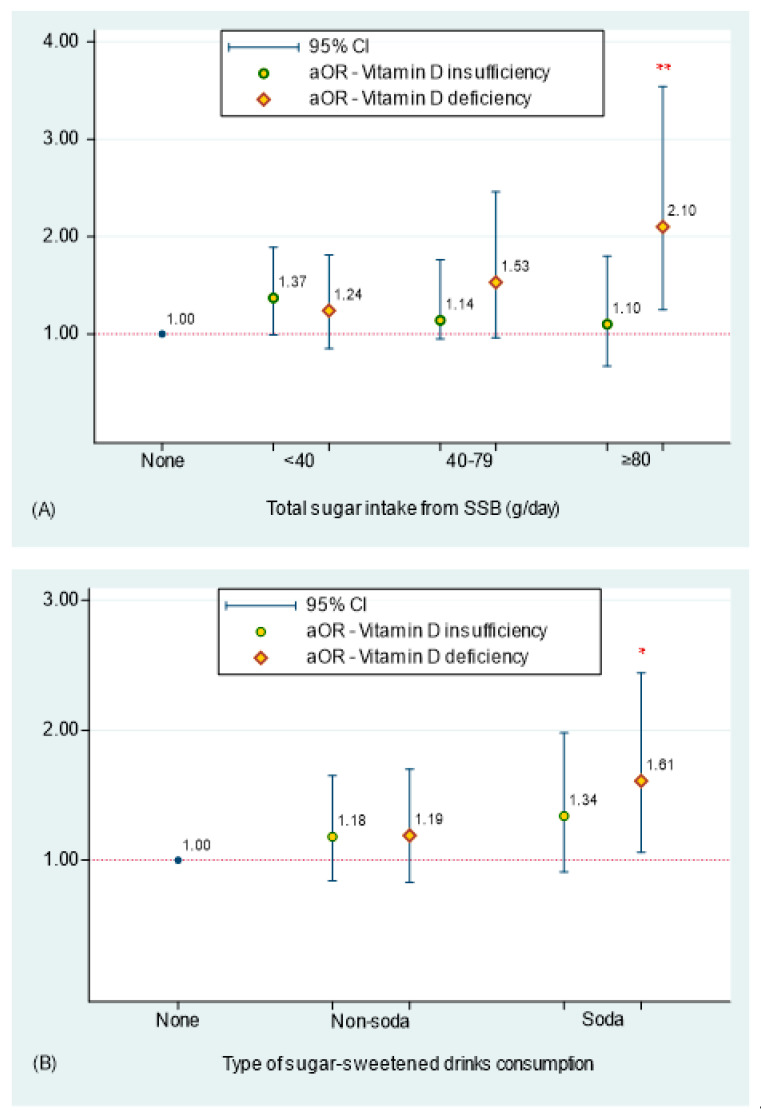
Adjusted odds ratios (aOR) and 95% confidence intervals (CI) of total sugar intake from sugar-sweetened beverage (SSB) in figure (**A**) and type of SSB consumption in figure (**B**) associated with risks of vitamin D insufficiency and deficiency were assessed under multinominal logistic regression models after adjusting for potential confounders and BMI status. Note: None, non-SSB consumers; Non-soda, sweetened tea/coffee and juice flavored SSB only; Soda, soda only and all types of SSB including soda. *, *p*-value = 0.030; ** *p*-value = 0.008.

Figure 2 illustrates the effect of total sugar intake from SSB and type of SSB consumption on vitamin D insufficiency and deficiency using multinomial regression models after adjusting for potential confounders and BMI, respectively. Compared to non-SSB consumers, a 2.10-fold increased risk of vitamin D deficiency was found in U.S. adults who consumed ≥80 g of sugar from total SSB per day (95%CI = 1.25, 3.54) (Figure 2A). Furthermore, a 1.61-fold higher risk of having vitamin D deficiency was observed in soda consumers when compared to non-SSB consumers (95%CI = 1.06, 2.44).

## 4. Discussion

This study’s findings revealed that total sugar intake from SSB consumption and BMI was an independent risk inversely associated with a lower 25(OH)D level, after controlling for potential confounders. When compared to non-SSB consumers, a higher risk of vitamin D deficiency was estimated in heavy SSB consumers and soda consumers. Furthermore, BMI may play a vital role in mediating the effect of high total sugar intake from SSB on decreased 25(OH)D levels in U.S. adults.

In the current study, a positive association between total sugar intake from SSB and prevalence of vitamin D insufficiency and deficiency was found. When compared to non-SSB consumers, an increased prevalence of vitamin D deficiency was observed in U.S. adults who were light, medium, and heavy SSB consumers (21.9% in light, 30.5% in medium, and 40.0% in heavy SSB consumers). In heavy SSB consumers, only 24.6% individuals had with normal vitamin D levels. An inverse association between amounts of sugar from SSB and level of 25(OH)D was still observed, even after adjusting for personal characteristics, medical conditions, and dietary-related factors. The current study further explored the effect of total sugar intake from SSB on the risk of vitamin D deficiency. A 2.10-times increased risk of vitamin D deficiency was estimated in U.S. adults who consumed ≥80 g of sugar from total SSB per day when compared to non-SSB-consumers. To date, the findings on the impact of SSB consumption on vitamin D concentrations are still controversial and limited. Few findings have reported that SSB intake, particularly carbonated SSB and juice, may affect concentrations of 25(OH)D, including one experimental study of rats [41], one cross-sectional study of children [26], one study of premenopausal women [25], one most recent study of patients with Hashimoto’s thyroiditis [27], and animal experimental animal study [42]. The study on premenopausal women found a significant negative association between cola intake and 25(OH)D concentrations, but there was no association between fruit juice consumption and 25(OH)D concentrations [25]. Another study on differences in 25(OH)D concentrations among obese and non-obese children found that obese children reported higher consumption of SSBs and lower 25(OH)D concentrations [26]. Additionally, one study found a minimal positive association between 25(OH)D concentrations and non-alcoholic drinks [27]. The results from this study differ from the literature but are possibly due to the non-alcoholic drink reported in the study’s FFQ being specific to the study’s country of origin, thus skewing the results. An animal study showed that significantly decreased plasma 25(OH)D was observed in rats fed with colas compared to those that received water only [41]. In the current study, we further evaluated the association between different types of SSB intake and vitamin D deficiency. SSB consumers were classified into non-soda and soda consumers. A higher prevalence of vitamin D deficiency was found in soda consumers. Additionally, a high prevalence of vitamin D deficiency was also found in soda consumers within SSB consumers (32.1% in soda consumers and 23.3% in non-soda consumers, *p* = 0.002). Furthermore, the association between different types of SSB consumption and vitamin D levels was investigated. Compared to non-SSB consumers, a 1.61-times higher risk of having vitamin D deficiency was observed in soda consumers. The first possible explanation for this finding is that carbonated beverages, such as colas, contain higher amounts of fructose and possibly influence vitamin D metabolism. Some evidence suggests that chronic fructose intake may negatively impact vitamin D metabolism in the liver and kidneys [43]. A 30–40% reduction in 25(OH)D levels was observed in rats fed with fructose compared to those fed with glucose [42]. Another possible reason is that the adverse effect on vitamin D and calcium metabolism may be influenced by the higher phosphoric acid intake from soda drinks [44,45].

A higher prevalence of insufficient vitamin D levels was found in individuals with a greater BMI in a population-based survey study [46]. Findings from one study that analyzed twenty-one cohorts revealed that BMI was positively associated with prevalence of vitamin D deficiency [19]. A previous study showed that obese populations had a 3.4 nmol/l decreased serum 25(OH)D when compared to those who were of normal weight [16]. Similarly, a higher prevalence of vitamin D deficiency was found in our study among subjects who were obese. Only 30.5% of obese individuals had sufficient 25(OH)D levels. Although the causal relationship between obesity and lower 25(OH)D is still controversial, a growing number of studies support the possible causal effect of obesity on lower 25(OH)D that may be explained by several factors, including dietary intake patterns, sunlight behavior, body composition, and biological mechanisms. A healthier diet consisting of an increased consumption of vegetables and whole grain products and higher levels of vitamin D is associated with a lower risk of poor metabolic health [16]. Obese populations may also engage in lower physical activity and exposure to insufficient sunlight compared to populations who are of normal weight [47]. Some possible biological mechanisms have been proposed to explain why the inverse association between serum 25(OH)D concentrations and BMI are found. First, the distribution of both serum vitamin D and 25(OH)D may be diluted in obese populations due to greater tissue volume, including serum, fat, muscle, liver, and other tissues [48]. An experimental study evaluated serum 25(OH)D levels after intaking vitamin D supplements between different BMI statuses. The findings showed that a lower increased serum 25(OH)D level was observed in obese populations compared to normal-weight populations after intake of vitamin D supplements [49]. Furthermore, obesity may decrease the bioavailability of vitamin D obtained from the diet or cutaneous synthesis [16].

Besides higher BMI status, several risk factors such as personal characteristics, lifestyle patterns, and daily dietary intake are important factors linked to serum vitamin D levels. Personal characteristics such as ethnicity, lifestyle behaviors, and socioeconomic status have been known to be associated with variations in 25(OH)D levels [15,16]. Dark skin pigmentation is linked to having lower 25(OH)D levels [50]. Older age, insufficient physical activity, and current smoking have also been found to be associated with low serum 25(OH)D concentrations [51,52]. Prior findings also suggest that socioeconomic status is related to poor diet quality and, further, lower serum 25(OH)D. In this study, similar findings revealed that a lower 25(OH)D level was found in individuals who were male, non-Hispanic Black, below the poverty income ratio, current smokers, and those who engaged in lower physical activity. In addition to sun exposure [53], vitamin D is also obtained via the diet, including foods and dietary supplements [16,54]. The current study indicated that a significantly lower prevalence of vitamin D insufficiency and deficiency was shown in individuals who consumed the recommended dietary allowances (RDA)-suggested daily values of vitamin D or calcium. This study also observed that individuals with self-reported medical conditions consumed higher levels of vitamin D compared to those who did not report any medical conditions (data not shown). This may possibly explain why the lowest prevalence of vitamin D deficiency was revealed in individuals with medical conditions. Therefore, demographic factors, cigarettes and alcohol use, physical activity status, personal medical conditions, and daily total intake of energy, sugar, vitamin D, and calcium were further adjusted. Our findings consistently showed that a significantly higher risk of vitamin D deficiency appeared in U.S. adults who consumed high amounts of sugar from SSB or soda drinks when compared with non-SSB consumers.

The overconsumption of SSB is well known to be associated with several poor health outcomes, in particular high body fat and obesity [22]. A few studies also have shown that carbonated SSB or juice intake may affect concentrations of 25(OH)D levels. Interestingly, we found that not only was BMI associated with decreased 25(OH)D levels, but sugar intake may also lower 25(OH)D levels. This is the first finding to reveal the negative association between sugar intake from all sweetened beverages and the risk of vitamin D deficiency in the general population. Whether BMI plays a role in the relationship between total sugar intake from SSB and lower 25(OH)D is unclear. In the current study, the effect of total sugar intake from SSB on decreased 25(OH)D levels was much smaller when the influence of BMI was taken into account. Therefore, a mediation analysis was further performed in the current study. We found that approximately 21.3% of the relationship between total sugar intake from SSB and 25(OH)D levels was significantly mediated by BMI. The total effect is about 1.27 times the direct effect. Our findings suggest that BMI plays an important role in the association between total sugar intake from SSB and decreased 25(OH)D levels in U.S. adults. Reduction of SSB intake is suggested, which may be beneficial for healthy BMI management and lower risk of vitamin D deficiency.

## 5. Limitation and Strengths

This study has some limitations. First, the effect of total sugar intake from SSB and decreased 25(OH)D was explained by BMI based on mediation analysis. Due to the fact that cross-sectional survey data were utilized in this study, causal inferences cannot be made. Second, some misclassifications of information dietary intake could not be eliminated due to self-reported 24-h recall interviews. However, the bias of misclassifications should be non-differential and minimally influence the current findings. Third, the effect of human genetic constitution and the chemical composition on 25-hydroxyvitamin D levels cannot be calculated in this study due to data limitation. Fourth, a public database was utilized to explore our study questions. Future studies are needed for confirmation by adequately designed studies with prospective data collection. Furthermore, multiple comparison justification was not performed in this study because this study only has two primary predictors (sugar intake from SSB and type of SSB intake) representing different dimensions of SSB intake. Except for these two SSB-related variables and BMI, other factors were treated as potential confounding factors. Regardless of these limitations, our study has several strengths. First, a large population-based survey’s data were utilized in this study, and our findings could represent the national U.S. population. Second, two-day 24-h dietary recall interviews were used to identify each type of SSB. Total sugar intake from SSB was further evaluated based on the USDA codes. Third, to our knowledge, this is the first study to demonstrate that BMI may be a mediator in the relationship between SSB intake and lower 25(OH)D levels in U.S. adults. Fourth, several important factors were related to 25(OH)D levels, such as total daily nutrient intake, including energy, vitamin D, and calcium, and physical activity as potential confounders.

## 6. Conclusions

In conclusion, high sugar consumption from SSB, regardless of additional nutrients or mineral components, and overweight/obesity are independently associated with lower 25(OH)D levels. Although some components may influence the vitamin D levels in the blood, an increased risk of vitamin D deficiency was found in either heavy SSB consumers or soda consumers when compared to non-SSB consumers. Furthermore, the current findings demonstrate that BMI may play a role as a mediator in the association between SSB intake and 25(OH)D levels.

## Figures and Tables

**Table 1:** Distribution of demographic factors and lifestyle patterns among 25-hydroxyvitamin D (ng/mL) status (normal, insufficiency, and deficiency).

Factors	25-Hydroxyvitamin D (ng/mL)					
	Mean ± se	*p*-Value	Normal	Insufficiency	Deficiency	*p*-Value
**Number of population ^1^**	N = 4505		N = 1465	N = 1687	N = 1353	
**Survey-weighted ^2^**	28.0 ± 0.5		38.1%	37.8%	24.1%	
**Personal characteristics**						
Age, years (mean ± se)	--	--	52.5 ± 0.7	44.9 ± 0.5	41.7 ± 0.9	<0.001
Gender		<0.001				<0.001
Male	26.6 ± 0.5		32.7%	42.1%	25.2%	
Female	29.4 ± 0.6		43.3%	33.6%	23.1%	
Ethnicity		<0.001				<0.001
Non-Hispanic White	30.7 ± 0.6		47.5%	38.1%	14.4%	
Non-Hispanic Black	20.2 ± 0.6		15.4%	26.0%	58.6%	
Mexican American	21.8 ± 1.0		14.9%	42.4%	42.7%	
Other Hispanic	25.6 ± 0.6		25.0%	50.4%	24.6%	
Other Race	24.9 ± 0.6		28.5%	37.3%	34.2%	
Ratio of family income to poverty		<0.001				<0.001
Below poverty	23.9 ± 0.7		24.7%	36.9%	38.3%	
1–1.99	26.2 ± 0.6		31.0%	39.4%	29.5%	
2–2.99	27.5 ± 0.9		32.4%	40.2%	27.4%	
3–3.00	28.5 ± 0.8		41.1%	37.9%	21.0%	
≥4	30.6 ± 0.8		48.6%	36.4%	15.0%	
**Lifestyle patterns**						
Cigarette use		<0.001				0.002
None	28.1 ± 0.6		38.8%	37.0%	24.2%	
Former	29.6 ± 0.5		41.7%	40.3%	18.0%	
Current	25.6 ± 0.8		31.3%	37.1%	31.7%	
Alcohol use		0.211				0.187
None	28.0 ± 0.7		35.4%	26.5%	28.1%	0.187
Light	28.3 ± 0.7		39.9%	37.6%	22.5%	
Heavy	27.0 ± 0.8		35.8%	41.2%	23.1%	
Physical activity (hour/week)		0.002				<0.001
Low	27.2 ± 0.4		35.0%	37.6%	27.3%	
Adequate	29.3 ± 0.8		43.2%	38.0%	18.8%	
Medical conditions ^3^		<0.001				<0.001
No	25.9 ± 0.6		28.7%	42.2%	29.2%	
Yes	29.7 ± 0.5		46.0%	34.1%	19.9%	

^1^ Raw number of participants in this study without adjusted for sample survey design. ^2^ Results were obtained after adjusting for sample weights and complex study design. ^3^ Medical conditions include asthma, arthritis, hypertension, heart diseases/attack, stroke, diabetes, angina, emphysema, thyroid problem, chronic bronchitis, and cancer/malignancy.

**Table 2:** Distribution of dietary patterns and physical examination indices among 25-hydroxyvitamin D (ng/mL) status (normal, insufficiency, and deficiency).

	25-Hydroxyvitamin D (ng/mL)					
	Mean ± se	*p*-Value	Normal	Insufficiency	Deficiency	*p*-Value
**Number of population ^1^**	N = 4505		N = 1465	N = 1687	N = 1353	
**Survey-weighted ^2^**	28.0 ± 0.5		38.1%	37.8%	24.1%	
**Daily dietary intake, mean** **± se**						
Total energy (kcal)	--	--	2073 ± 19	2081 ± 37	2157 ± 34	0.136
Total sugar (g)	--	--	106 ± 2.1	106 ± 2.0	114 ± 3.8	0.094
**Total nutrients intake ^3^**						
Vitamin D (D2 + D3) (μg)		<0.001				<0.001
Sufficient	34.8 ± 0.5		63.4%	30.2%	6.4%	
Insufficient	25.1 ± 0.5		27.4%	41.0%	31.6%	
Calcium (mg)		<0.001				<0.001
Sufficient	29.7 ± 0.6		45.3%	36.3%	18.4%	
Insufficient	26.4 ± 0.5		31.6%	39.1%	29.3%	
**SSB-related factors**						
Sugar intake from SSB (g), mean ± se	--	--	26.0 ± 2.9	36.1 ± 1.8	52.7 ± 3.7	<0.001
Total sugar intake from SSB per day		<0.001				<0.001
Non-SSB consumers	30.3 ± 0.6		47.1%	35.7%	17.1%	
<40 g	28.1 ± 0.7		36.0%	42.1%	21.9%	
40–79 g	25.9 ± 0.8		31.2%	38.4%	30.5%	
≥80 g	23.7 ± 0.5		24.6%	35.4%	40.0%	
Type of soda consumption (n = 2742)		<0.001				0.002
Non-soda SSB consumers ^4^	28.5 ± 0.7		38.8%	37.8%	23.3%	
Soda consumers ^5^	25.2 ± 0.6		28.0%	40.0%	32.1%	
**Body mass index (kg/m^2^)**		0.003				<0.001
Normal/under weight	29.9 ± 0.8		46.9%	32.4%	20.7%	
Overweight	28.3 ± 0.6		38.7%	40.4%	20.9%	
Obesity	26.1 ± 0.6		30.5%	39.8%	29.7%	
**Clinical biomarker**						
Alkaline phosphatase (IU/L)		0.880				0.582
Normal	28.0 ± 0.5		38.1%	37.9%	24.0%	
Abnormal	27.4 ± 3.9		39.1%	26.6%	34.3%	
Total triglycerides (mg/dL)		0.123				0.379
Normal	28.2 ± 0.6		39.2%	37.0%	23.8%	
Higher	27.6 ± 0.6		36.5%	39.0%	24.5%	
Phosphorus (mg/dL)		0.230				0.682
Normal	27.8 ± 0.6		37.8%	37.9%	24.3%	
Abnormal	29.5 ± 1.2		40.8%	36.9%	22.4%	

^1^ Raw number of participants in this study without adjusted for sample survey design. ^2^ Results were obtained after adjusting for sample weights and complex study design. ^3^ Daily total vitamin D and calcium intake from diet and supplements was categorized based on RDA suggestion, respectively. ^4^ Non-soda consumers included individuals who consumed only fruit-flavored sweetened drinks or sweetened tea/coffee. ^5^ Soda consumers included individuals who consumed either soda only or who consumed any type of SSB, including soda.

**Table 3:** The association between total sugar intake from SSB, BMI, and 25-hydroxyvitamin D (ng/mL) levels.

	25-Hydroxyvitamin D Levels (ng/mL)			
	Model 1		Model 2	
	aDiff	(95%CI)	aDiff	(95%CI)
**Total sugar intake from SSB**				
Non-SSB consumers	Ref		Ref	
<40 g	−1.07	(−2.42, 0.27)	−1.02	(−2.32, 0.28)
40–79 g	−1.83	(−3.23, −0.43) *	−1.42	(−2.88, 0.03)
≥80 g	−2.88	(−4.33, −1.44) ***	−2.53	(−3.93, −1.12) **
**Body mass index (kg/m^2^)**				
Normal/under weight	--	--	Ref	
Overweight	--	--	−1.58	(−2.95, −0.21) *
Obesity	--	--	−3.68	(−4.77, −2.59) ***

SSB, sugar-sweetened beverage; BMI, body mass index; Ref, reference group (non-SSB consumers). Model 1 represents adjusted differences (aDiff) that were estimated after adjusting for demographic factors, cigarettes and alcohol use, physical activity status, personal medical conditions, and dietary patterns (daily total intake of energy, sugar, vitamin D, and calcium). Model 2 represents adjusted differences (aDiff) that were estimated after adjusting for the covariates in model 1 and additionally for BMI. *, *p*-value < 0.05; **, *p*-value < 0.01; ***, *p* ≤ 0.001.

**Table 4:** The association between type of SSB intake, BMI, and 25-hydroxyvitamin D (ng/mL) levels.

	25-Hydroxyvitamin D Levels (ng/mL)			
	Model 1		Model 2	
	aDiff	(95%CI)	aDiff	(95%CI)
**Type of SSB intake**				
Non-SSB consumers	Ref		Ref	
Non-soda SSB ^1^	−0.50	(−1.88, 0.88)	−0.33	(−1.78, 1.11)
Soda drinks ^2^	−2.14	(−3.37, −0.90) **	−1.92	(−3.14, −0.70) **
**Body mass index (kg/m^2^)**				
Normal/under weight	--	--	Ref	
Overweight	--	--	−1.60	(−3.03, −0.16) *
Obesity	--	--	−0.71	(−4.84, −2.58) ***

SSB, sugar-sweetened beverage; BMI, body mass index; Ref, reference group (non-SSB consumers). Model 1 represents adjusted differences (aDiff) that were estimated after adjusting for demographic factors, cigarettes and alcohol use, physical activity status, personal medical conditions, and dietary patterns (daily total intake of energy, sugar, vitamin D, and calcium). Model 2 represents adjusted differences (aDiff) that were estimated after adjusting for the covariates in model 1 and additionally for BMI. ^1^ Non-soda consumers included individuals who consumed only fruit-flavored sweetened drink or sweetened tea/coffee. ^2^ Soda consumers included individuals who consumed either soda only or who consumed any type of SSB, including soda.*, *p*-value < 0.05; **, *p*-value < 0.01; ***, *p* ≤ 0.001.

## Data Availability

Not applicable.
